# Plant Defenses Against Pests Driven by a Bidirectional Promoter

**DOI:** 10.3389/fpls.2019.00930

**Published:** 2019-07-17

**Authors:** Ana Arnaiz, Manuel Martinez, Pablo Gonzalez-Melendi, Vojislava Grbic, Isabel Diaz, M. Estrella Santamaria

**Affiliations:** ^1^Centro de Biotecnología y Genómica de Plantas, Instituto Nacional de Investigación y Tecnología Agraria y Alimentaria, Universidad Politécnica de Madrid, Madrid, Spain; ^2^Departamento de Biotecnología-Biología Vegetal, Escuela Técnica Superior de Ingeniería Agronómica, Alimentaria y de Biosistemas, Universidad Politécnica de Madrid, Madrid, Spain; ^3^Department of Biology, The University of Western Ontario, London, ON, Canada

**Keywords:** bidirectional promoter, *cis*-elements, defense genes, inducible promoter, reporter gene expression, *Tetranychus urticae*, *Pieris brassicae*

## Abstract

The plant defense responses to pests results in the synchronized change of a complex network of interconnected genes and signaling pathways. An essential part of this process is mediated by the binding of transcription factors to the specific responsive *cis*-elements within in the promoters of phytophagous-responsive genes. In this work, it is reported the identification and characterization of a bidirectional promoter that simultaneously co-regulate two divergent genes, *At5g10300* and *At5g10290*, upon arthropod feeding. Computational analysis identified the presence of *cis*-elements within the intergenic region between two loci, mainly from the DOF but also from the AP2/ERF, Golden 2-like and bHLH families. The function of the bidirectional promoter was analyzed using two enhanced variants of the *GFP* and *CherryFP* reporter genes, in both orientations, in transient tobacco and stably transformed Arabidopsis plants. Promoter activity was tested in response to feeding of *Tetranychus urticae* and *Pieris brassicae*, as well as wounding, flagellin and chitin treatments. Using RT-qPCR assays and confocal microscopy, it was shown that all treatments resulted in the induction of both reporter genes. Furthermore, our findings revealed the asymmetric character of the promoter with stronger activity in the forward than in the reverse orientation. This study provides an example of a bidirectional promoter with a strong potential to be used in plant biotechnology in pest control that requires stacking of the defense genes.

## Introduction

A bidirectional promoter is the intergenic region between two divergent genes located on complementary strands of the DNA which drives their coordinated transcription in opposite directions. These promoters control of the expression of gene pairs usually involved in the same or in related physiological processes (Mitra et al., 2009; Singh et al., 2009; Oropeza-Aburto et al., 2017). Most of the known bidirectional promoters have been found during the research of single genes, but the development of sequencing technologies and bioinformatics tools discovered that bidirectional nuclear gene promoters are very abundant in eukaryotes, including plants (Wang et al., 2009, 2016; Dhadi et al., 2013). In a first search of bidirectional promoters in the Arabidopsis genome, Wang et al. (2009) identified near 2,500 gene shared intergenic regions, enriched in regulatory elements essential for transcription. The characterization of some of them showed that the majority drove gene pairs within same functional category. Comparative analysis of bidirectional promoters, performed in rice, Arabidopsis and Populus (Dhadi et al., 2009) and in three grass species such as maize, sorghum, and Brachypodium (Krom and Ramakrishna, 2010) provided very useful information about common *cis*-elements, regulatory mechanisms, co-expression and evolutionary dynamics.

The first characterized bidirectional promoter from plants was identified in maize. Shwarz et al. (1981) found that two genes overlapped by few nucleotides in the maize chloroplast chromosome were transcribed divergently from complementary DNA strands. Since then, bi-directionality of promoters controlling genes in a head-to-head fashion has been described in many species. Some intergenic regions drive an equal expression of the two genes in both directions. One example is the co-expression of two chymotrypsin protease inhibitor encoding genes from rice, *OCPI2* and *OCPI1*, with 1,126 bp distance between their transcription initiation sites in reverse orientations. *OCPI2* and *OCP1* transcripts were coordinately regulated and induced in an almost identical manner by salt, ABA, low temperature and mechanical wounding treatments in roots and shoot tissues of seedlings (Singh et al., 2009). In other cases, the transcription of some genes under a bidirectional promoter is dependent on the orientation. An example is the tissue-expression profile shown by the two divergent *At4g35985* and *At4g35987* Arabidopsis genes, encoding a senescence associated protein and a calmodulin methyltransferase, respectively, that shared a 1,258 bp intergenic region. While the genes were highly expressed in apical meristem and shoot and root tips in one orientation, in the opposite strand they were very active in mature tissues (Banerjee et al., 2013). Expression assays of this bidirectional promoter fused to Green Fluorescence Protein (GFP) and β-glucuronidase (GUS) reporter genes in both orientations in stable transformed Arabidopsis and tobacco plants corroborated the tissue specificity, and pointed-out a specific up-regulation under salt and pathogen stresses dependent on the orientation, strength and the exposure time of the stress. Generally, most bidirectional promoters direct the asymmetric transcription of the gene pair under their control, even when they are expressed in the same plant tissue. This is the case of two Arabidopsis genes, *At1g71850* (ubiquitin carboxyl-terminal hydrolase) and *At1g71860* (tyrosine-specific protein phosphatase) separated by a 347 bp intergenic sequence which exhibited an orientation-dependent expression profile (Liu et al., 2015). Likewise, the intergenic region of the maize defensin-like protein genes *Def1* and *Def2* determined embryo- and aleurone-specific expression but displayed asymmetric activity (Liu et al., 2016). The deletion of the *cis*-acting elements contained in this promoter evidenced that the transcription polarity and differential strength were dependent on the presence of specific *cis*-motifs. Recently, the functional analysis of a bidirectional promoter (1,429 bp) shared by a gene pair from *Gossypium hirsutum*, *Ghrack1* (receptor for activated C kinase1), and *Ghhrf1* (E3 ubiquitin-protein ligase), has also showed an orientation dependence (Yang et al., 2018). Using series of expression vectors containing *GUS* and *GFP* reporter genes in forward and reverse orientations of the promoter to transform Arabidopsis and tobacco, they found differences on spatial and temporal gene expression controlled by the promoter orientation.

The selection of a promoter is essential for the successful expression for multigene engineering strategies. Bidirectional promoters offer certain advantages including: (i) coordinated expression of two genes; (ii) reduction in time-consuming construction of expression vectors; (iii) prevention of silencing of multigene expression based on the non-repetitive use of the same unidirectional promoter; (iv) stability of the DNA based on the reduced amount of foreign DNA in genetically engineered plants. In this context, to transform a unidirectional promoter into a bidirectional one, or to design artificial bidirectional promoters has been considered as an alternative adapted to transcribe genes under specific physiological and environmental stimuli. Current bioinformatics tools, databases and technologies allow the design and the introduction of *cis*-elements in bidirectional promoters as an artwork of *cis*-engineering (Mehrotra et al., 2011). Moreover, changes in the core and flanking sequences of the *cis*-motifs may lead to alterations in the gene expression in both native and synthetic promoters (Zhang et al., 2019). Xie et al. (2001) created the first artificial plant bidirectional promoter based on the 35S minimal promoter (51 bp from transcription start point) from the cauliflower mosaic virus (CaMV) by fusing the 35S mini-promoter at its 5’ end in the opposite orientation. The same strategy was used by Zheng et al. (2011) to generate a bidirectional methyl jasmonate-inducible promoter derived from poplar. Kumar et al. (2015) combined the construction of bidirectional promoters and a biscistronic approach for coordinated multi-gene expression in maize. They integrated the polyprotein processing T2A ACD sequence from *Thosea asigna* virus into a synthetic bidirectional promoter derived from the *Ubiquitin-1* promoter from *Zea mays* to improve transgene stacking strategy. Expression analysis of transgenic maize plants confirmed the coordinated expression of four genes conferring insect and herbicide resistance based on this combined approach. More recently, Vogl et al. (2018) generated a library of 168 synthetic bidirectional promoters in the yeast *Pichia pastoris* by using its natural histone promoters as an engineered template. The rapid screening of expression profiles to optimize gene co-expression suggested the potential of this approach for other eukaryotes, including plants.

In this work, we report the identification and characterization of a bidirectional promoter shared by two divergent genes, *At5g10300* and *At5g10290*, simultaneously transcribed and induced by the two-spotted spider mite feeding. The promoter function was tested by fusing *mGFP* and *mCherryFP* reporter genes in both orientations in transiently agroinfiltrated tobacco leaves and in stably transformed Arabidopsis plants. These plants were used to analyze their responses to arthropod feeding and other related defense-treatments by RT-qPCR assays and confocal microscopy.

## Materials and Methods

### *In silico* Analysis of the Bifunctional Promoter

The VISTA plots of multiple genomic comparisons of Brassicaceae species different from *Arabidopsis thaliana* were visualized in the VISTA-Point tool using the pairwise precomputed alignments from the VISTA platform (Frazer et al., 2004). The VISTA plots of pairwise comparisons of putative bifunctional promoter sequences of *Arabidopsis lyrata* and *Arabidopsis halleri* species to *A. thaliana* were generated by the zPicture tool (Ovcharenko et al., 2004) using the sequences retrieved from the Araport11 annotation (Cheng et al., 2017). Multiple sequence alignment was prepared using the MUSCLE program in the SeaView4 platform (Gouy et al., 2010). Transcription factors sites were obtained using the binding site prediction tool in the PlantRegMap and the PlantTFDB 4.0 database (Jin et al., 2017).

### Plant Material and Growth Conditions

*Arabidopsis thaliana* Columbia (Col-0) plants were used as wild-type plants. *A. thaliana* T-DNA mutants (Salk_022713C and Salk_015889C) were obtained from the Arabidopsis Biological Resource Centre and the European Arabidopsis Stock Centre (NASC).^1^ For *in vitro* growth, seeds were surface sterilized with 70% (V/V) ethanol for 2 min, followed by incubation in a solution containing 5% (V/V) sodium hypochlorite and 5% (W/V) SDS for 10 min, and then washed with sterilized distilled H_2_O. Seeds were plated onto Petri dishes containing 1/2 MS semisolid medium (Sigma-Aldrich), adjusted to pH 5.7 with KOH. For soil growth, a mixture of peat moss and vermiculite (3:2, V/V) was used. Sterilized seeds were stratified in the dark at 4^∘^C for 5 days. Plates and pots were grown in growth chambers (Sanyo MLR-351-H, Sanyo, Japan) under control conditions (23 ± 1^∘^C, >70% relative humidity and a 16^∘^h/8^∘^h day/night photoperiod).

*Nicotiana benthamiana* plants were grown in greenhouse under controlled conditions (22 ± 1°C, >70% relative humidity and a 16h/8h day/night photoperiod).

### Phytophagous Arthropod Maintenance

A colony of the two-spotted spider mite *Tetranychus urticae*, London strain (Acari: *Tetranychidae*), kindly provided by Dr. Miodrag Grbic (Department of Biology, Western Ontario University, Canada), was reared on beans (*Phaseolus vulgaris*) and maintained in growth chambers (Sanyo MLR-351-H, Sanyo, Japan) at 25 ± 1^∘^C, >70% relative humidity and a 16^∘^h/8^∘^h day/night photoperiod. A *Pieris brassicae* colony supplied by Prof. Dr. Marcel Dicke and Dr. Pieter Rouweler (Laboratory of Entomology, Wageningen University, Netherlands), was reared on Brussels sprouts (*Brassica oleracea* L. var. *gemmifera*) and maintained on growth chambers (Sanyo MLR-350-H, Sanyo, Japan) at 21 ± 1^∘^C, 50% relative humidity and a 16^∘^h/8^∘^h day/night photoperiod.

### Construction of Bidirectional Promoter-Reporter Gene Fusions and Plant Transformation

The intergenic region, between *At5g10290* and *At5g10300* genes (named in figures BP), was amplified using *A. thaliana* Col-0 genomic DNA as a template following the design schematized in Supplementary Figure 2 and the primers indicated in Supplementary Table 1. The *mCherryFP* ORF was amplified from the psmRS-mCherryFP plasmid (Barrero-Sicilia et al., 2011). PCRs were performed with Phusion Hot Start II High Fidelity DNA Polymerase according to the manufacturer’s instructions (Thermo Scientific), under the following reaction conditions: 30 s at 98^∘^C, followed by 30 cycles of 10 s at 98^∘^C, 20 s at 55^∘^C for 25 s (bidirectional promoter) or 53^∘^C for 20 s (*mCherryFP*) at 72^∘^C, and 5 min at 72^∘^C. The target DNA fragments of the forward and reverse orientations of the bidirectional promoter and the *mCherryFP* gene were ligated into the cloning vector pENTRY3C (Invitrogen). The *mCherryFP::BP* fusion were inserted in the pmdc107 vector containing the *mGFP* gene driven by a minimal promoter (Curtis and Grossniklaus, 2003), by homologous recombination using LR Clonase II enzyme mix (Invitrogen). Resultant fusion plasmids mCherryFP::BP::GFP and GFP::BP::mCherryFP, enclosed the two reporter genes driven by the BP in both orientations. The pmdc107, named here pmdc107ø, was used as control vector for all transformation events.

The fusion-constructs were first introduced into the *Agrobacterium tumefaciens* strain C58C1 Rif (*GV3101*) by using the freeze-thaw method (Weigel and Glazebrook, 2002). Then, 3-week old *N. benthamiana* leaves were transiently transformed by Agroinfiltration as described Santamaria et al. (2019), with the fusion-constructs mCherryFP::BP::mGFP, mGFP::BP::mCherryFP and the control pmdc107ø vector. Transient assays were performed after 3 and 4 days of infiltration (dpi). To generate stable transgenic plants, the fusion-constructs and the control vector pmdc107ø were also introduced into *A. thaliana* Col-0 plants using the *Agrobacterium* floral dip method (Clough and Bent, 1998). Transformants were selected on MS medium supplemented with 40 mg/L hygromicin. Homozygous lines with one single transgene copy coming from independent transformation events were selected from the T3 generation.

### Plant Infestation Assays

To analyse the phenotype of the promoter T-DNA insertion lines in response to spider mites, infestation assays were performed. Three-week-old Arabidopsis T-DNA Salk lines and Col-0 control plants were infested with 20 *T. urticae* female adults per plant. After 4 days of infestation, leaf damage was assessed by scanning the entire rosette (HP Scanjet 5590 Digital Flatbed Scanner series), according to Cazaux et al. (2014). Leaf damage was calculated in mm^2^ using Adobe Photoshop CS software. Six replicates were used for each genotype. The experiment was carried out in duplicate. To determine how the bidirectional promoter controls the expression of the two reporter genes in both orientations, feeding assays were performed with *T. urticae* and *P. brassicae*. Twenty spider mite female adults or five freshly hatched neonate caterpillar were placed on 3-week old Arabidopsis plants (pmdc107ø control and mCherryFP::BP::mGFP, mGFP::BP::mCherryFP lines), allowing a continuous feeding for 24^∘^h, in growth chambers (Sanyo MLR-351-H, Sanyo, Japan) at 25 ± 1^∘^C, >70% relative humidity and a 16^∘^h/8^∘^h day/night photoperiod. Six replicates of each plant genotype were performed per treatment and control. From these six plants, three were collected and frozen in liquid N_2_ for RT-qPCR and the other three plants were observed under the confocal microscopy. All the experiments were repeated three times, as is indicated in Supplementary Figure 3.

### Wounding, Flagellin, and Chitin Treatments

Arabidopsis plants (pmdc107ø control and mCherryFP::BP:: mGFP, mGFP::BP::mCherryFP transgenic lines) were also used to analyse the two reporter gene expression in response to mechanical wounding, flagellin (Flg22) and chitin treatments. For wounding, approximately 20% of the leaf area of two leaves, of 3-week old Arabidopsis plants, were pierced with a needle and their leaves were collected 1^∘^h after. In the case of the Flg22 treatment, plants were sprayed with 200 μl of the flagellin-derived Flg22 peptide (AnaSpec) at a final concentration of 5 μM. Control plants were sprayed with the same amount of water. Plants were collected after 12^∘^h of treatment. Chitin stock solution were prepared by dissolving chitin from shrimp shells (Sigma-Aldrich) in DMSO at a concentration of 25 mg/mL and subsequently diluted in H_2_O to a 100 μg/mL concentration for the experiment. The solvent used to solubilize the elicitor (DMSO) was used as control treatment at a final concentration of 0.4% (V/V). Plants were sprayed using 200 μl per plant and leaves were collected after 24^∘^h of treatment. Growth conditions were the same mentioned above and experimental design and replicates were done according to Supplementary Figure 3.

### Nucleic Acid Analysis

Genomic DNA was isolated from Arabidopsis T-DNA insertion lines and Col-0 plants, essentially as described by Sambrook and Russell (2001). The T-DNA homozygous status and gene expression levels of the Salk lines was analyzed by conventional PCR and quantitative PCR (RT-qPCR) analyses (Supplementary Figures 4A–C). Conventional PCR conditions were: 3 min at 94^∘^C, followed by 30 cycles for 30 s at 94^∘^C, 40 s at 55^∘^C, and 1 min 20 s at 72^∘^C, and finally 7 min at 72^∘^C.

For RT-qPCR studies, total RNA was isolated from 3-week old rosettes from Arabidopsis T-DNA insertion and Col-0 plants, and from different tissues (seedlings, rosettes, roots and mature seeds) of Arabidopsis plants (pmdc107ø control and mCherryFP::BP::mGFP, mGFP::BP::mCherryFP transgenic lines) after different treatments. Total RNA from seedlings, 3-weel-old-rosettes and roots was extracted by the phenol/chloroform method, followed by precipitation with 8 M LiCl according to Oñate-Sánchez and Vicente-Carbajosa (2008). RNA form mature seeds was isolated by the hot borate method described by Santamaria et al. (2010). Regarding *N. benthamiana* assays, total RNA was extracted from plants agroinfiltrated with the control vector pmdc107ø, the mGFP::BP::mCherryFP and the mCherryFP::BP::mGFP constructs, by the TRIZOL reagent following manufacturer instructions (Ambion Austin TX). Complementary DNAs (cDNAs) were synthesized from 2 μg of RNA using the Revert Aid^TM^ H Minus First Strand cDNA Synthesis Kit (Fermentas) as indicated manufacturer’s instructions. The RT-qPCR conditions used were 40 cycles with 15 s at 95^∘^C, 1 min at 60^∘^C and 5 s at 65°C using LightCycler 480 SYBR Green I Master (Roche). RT-qPCR was performed in three samples coming from three independent experiments as previously described by Santamaria et al. (2012), using a SYBR Green Detection System (Roche) and the LightCycler480 Software release 1.5.0 SP4 (Roche). mRNA quantification, expressed as relative expression levels (2^–*dCt*^), was normalized to ubiquitin or actin for Arabidopsis and Nicotiana samples, respectively. For mRNA quantification referred as fold change (2^–*ddCt*^) in Arabidopsis samples, ubiquitin as housekeeping gene and untreated control sample as internal calibrator were used (Livak and Schmittgen, 2001). Specific primers were designed through PRIMER3.^2^ Primer sequences are listed in Supplementary Table 1.

### Confocal Images

All images were obtained with the TCS-SP8 confocal microscope (Leica). For transient expression experiments, *N. benthamiana* leaves were observed under the microscope after 3 and 4 days of the agroinfiltration (dpi). Confocal images of Arabidopsis leaves were taken after arthropod feeding assays, mechanical wounding and elicitor treatments in stable transformed Arabidopsis plants. Three independent leaves of each genotype, for control and treatments, were observed. In all experiments, mGFP and mCherryFP signals were sequentially acquired using the following settings: mGFP, excitation 488 nm and emission 491–550 nm; mCherryFP, excitation 561 nm, emission 591–638 nm.

### Statistical Analysis

The statistical analysis was performed using GraphPadPrismv6.01 and IBM SPSSv25 statistics. In order to perform the proper statistical analysis, normality and homoscedasticity of the data were previously analyzed. In the case of the foliar damage, data fulfill both assumptions so, one-way ANOVA followed by Tukey’s multiple comparisons test were used. In the case of the gene expression, analysis generalized linear models stats was performed. Wald χ^2^ followed by LSD test was applied in all the RT-qPCR experiments. In figures, significant differences (*P* < 0.05) among transgenic lines are reported with different letters.

## Results

### Structure and Sequence Analysis of an Arabidopsis Intergenic Region Between Two Genes Involved in Defense to *T. urticae*

**FIGURE 1:**
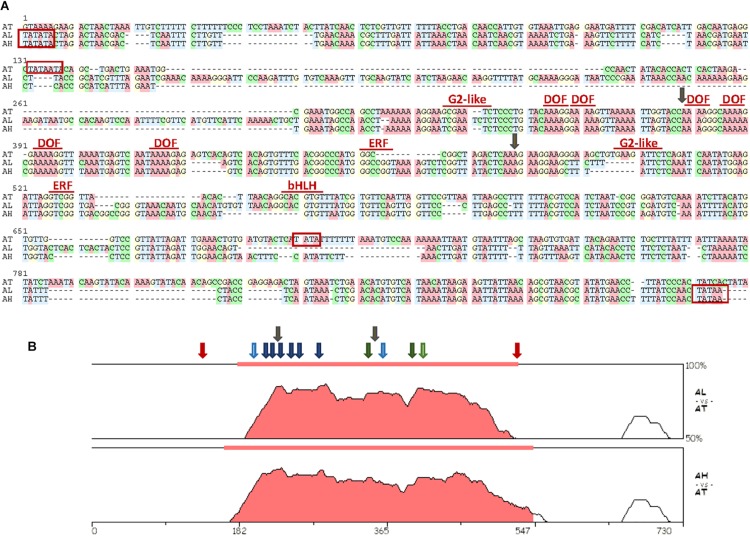
Sequence characteristics of the region spanning the interval between putative TATA sequences in the bifunctional promoter. **(A)** Alignment of the nucleotide sequences of the selected interval. Names of transcription factor families mark their putative binding sites. TATA predicted sites are boxed in red. Insertion points for the T-DNA lines used are marked by gray arrows. Transcription factors sites were obtained using the binding site prediction tool in the PlantRegMap and the PlantTFDB 4.0 database. Alignment was prepared using the MUSCLE program in the SeaView4 platform. **(B)** The VISTA plots of pair-wise comparisons of putative bifunctional promoter sequences of *A. lyrata* and *A. halleri* species to *A. thaliana* as generated by the zPicture tool. Filled portions of the graphs indicate conservation of more than 50% with a width of at least 100 bp. Approximate locations of conserved binding sites for transcription factors are marked by arrows (dark blue, DOF; light blue, G2-like; dark green, ERF; light green, bHLH). TATA predicted sites for *A. thaliana* are marked by red arrows. Insertion points for the T-DNA lines used are marked by gray arrows.

Zhurov et al. (2014) analyzed the transcriptional response to spider mite infestation in resistant Bla-2 and susceptible Kondara Arabidopsis accessions. From this analysis, many differentially expressed genes were identified. Looking at the genomic context of some genes up-regulated in Bla-2 upon mite feeding, we found that one induced gene, *At5g10300*, shared its potential promoter region with a second gene, *At5g10290*, annotated in the opposite direction. The *At5g10300* and *At5g10290* genes encoded a hydroxynitrile lyase and a LRR receptor kinase, respectively. A region of only 645 bp was found between the putative transcription start sites of both genes. To get a first insight on the relevance of this genomic arrangement, a genomic comparison of this region among Brassicaceae species was performed (Supplementary Figure 1). From this comparison, a strong conservation between the genes flanking the promoter was observed (Supplementary Figure 1A). However, conservation in the promoter sequences was mainly restricted to the promoters from Arabidopsis species, with minor similarities with the promoters of Brassicaceae species from other genera (Supplementary Figure 1B). Then, the nucleotide sequences of the promoters from the three Arabidopsis species were aligned to detect conserved functional motifs (Figure 1A). The putative location of TATA boxes was dissimilar between *A. thaliana* and the other two species. The TATA motifs were located more internally in *A. thaliana* which reflected in the lower number of bp flanked by the TATA motif in this species (385 bp) than in *A. lyrata* and *A. halleri* (764 and 602 bp, respectively). In any case, the strongest conserved region was located in the sequence flanked by the most internal TATA boxes (Figure 1B). Many binding sequences for transcription factors (TFs) were identified in this region. Among them, it is remarkable the presence of several conserved DOF-binding motifs (AAAG/CTTT), as well as some *cis*-elements potentially recognized by defense-related TFs from the AP2/ERF (GCC box), Golden 2-like (GAATCT) and basic Helix-Loop-Helix, bHLH (CACGTG) TF families.

### Expression Analysis of *At5g10290* and *At5g10300* Genes and Responses to Spider Mite Feeding

To determine whether the bidirectional promoter controls the simultaneous transcription of the gene pair *At5g10290* and *At5g10300*, RT-qPCR assays were carried out in Arabidopsis rosettes of Col-0 plants and two T-DNA insertion lines. Results demonstrated that both genes were transcribed at the same time but the relative transcript abundance of the *At5g10290* gene was higher compared to the relative expression of *At5g10300* gene in the opposite strand (Supplementary Figure 4D). The alleles generated by the insertion of T-DNA in the promoter (Salk_015889C and Salk_022713C lines) corroborated the promoter bi-directionality. A reduction in the expression levels of the two divergent genes in the mutant lines were observed in comparison to the expression found in Col-0 plants. These data indicated the double knock-down character for *At5g10290* and *At5g10300* genes of these mutant lines.

**FIGURE 2:**
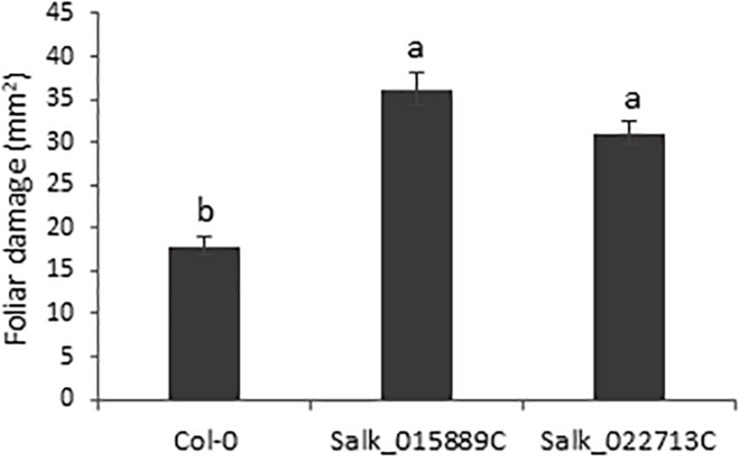
Plant damage of Arabidopsis Col-0 and T-DNA insertion lines infested with *T. urticae.* Damaged foliar area was measured after 4 days of spider mite infestation. Data are means ± SE of six replicates. Different letters indicate significant differences (*P* < 0.05, One-way ANOVA followed by Tukey’s multiple comparisons test).

The homozygous T-DNA insertion lines and the Col-0 plants were used to test the involvement of the bidirectional promoter in the plant response to *T. urticae* feeding. Plants were infested with *T. urticae* adults and leaf damage was quantified 4 days after infestation. The damage intensity, measured as the chlorotic foliar area, was approximately twofold higher in the knock-down lines than the damage quantified in Col-0 plants after mite infestation (Figure 2). Thus, greater leaf damage parallel to a lower pair gene expression, reflected not only the potential defensive role of the genes located on the opposite strands but also the precise control of the bidirectional promoter.

### Functional Analysis of the Bidirectional Promoter in Transiently Transformed *N. benthamiana* Plants

**FIGURE 3:**
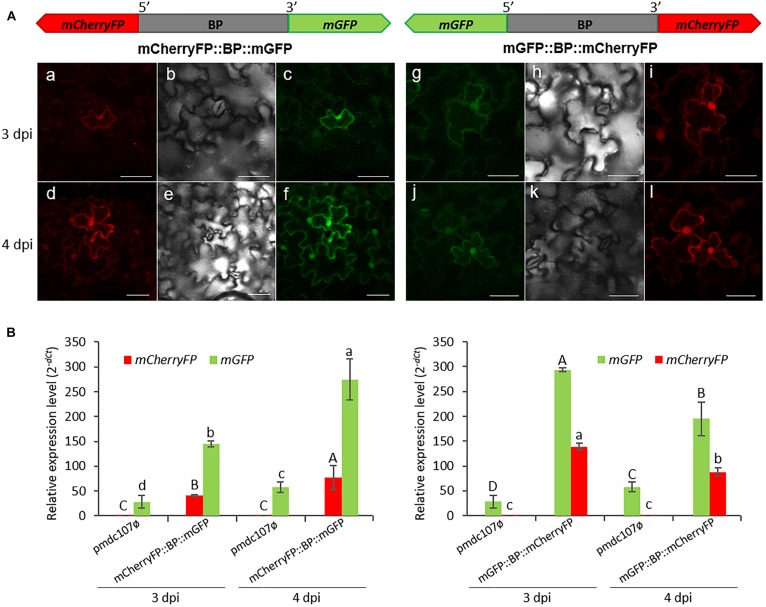
Schematic representation of fusion-constructs and transient expression assays in *N. benthamiana* leaves. **(A)** Scheme of *mCherryFP* and *mGFP* genes under the bidirectional promoter (BP) and confocal stacks spanning tobacco epidermal cells of transient transformed leaves at 3 and 4 post-infiltration days (dpi). Projections are from mCherryFP **(a,d,i,l)**, mGFP **(c,f,g,j)** and the corresponding Nomarski snap-shots **(b,e,h,k)**. Scale bars represent 50 μm. **(B)** Relative expression levels (2^–*dCt*^) of *mCherryFP* and *mGFP* genes in agroinfiltrated *N. benthamiana* leaves with the control vector (pmdc107ø) or with the constructs including the bidirectional promoter fused to the two reporter genes. Data are means ± SE of three replicates. Different letters indicate significant differences (*P* < 0.05 Wald χ^2^ followed by LSD test). Primers used for this experiment are listed in Supplementary Table 1.

The intergenic region between *At5g10290* and *At5g10300* genes was used to make transcriptional fusions to *mGFP* and *mCherryFP* genes in the opposite orientations. Transient expression assays in agroinfiltrated *N. benthamiana* leaves revealed that both reporter genes were co-expressed and the emitted fluorescence was localized in the nuclei, membrane and some cytoplasmic areas, independent on the orientation (Figure 3A). No detectable florescence was found under the confocal microscope when the vector pmdc107ø was used (data not shown).

Transcript analysis of the reporter genes performed by RT-qPCR assays, at 3 and 4 dpi, corroborated the simultaneous expression of both genes (Figure 3B). The *mGFP* gene presented higher expression levels than the *mCherryFP* gene, independently on the gene orientation. Basal expression levels of the *mGFP* gene were obtained with the pmdc107ø control vector due to the presence of the minimal promoter contained in the control plasmid. As expected, no detection of the *mCherryFP* gene was found.

### Functional Analysis of the Bidirectional Promoter in Stably Transformed *A. thaliana* Plants

To investigate the role of the bidirectional promoter in response to different stresses, the same fusion-constructs were used to stably transform *A. thaliana* plants. The characterization of the homozygous lines with one single transgene copy coming from independent transformation events allowed the selection of lines (1.1 and 2.4 for the mGFP::BP::mCherryFP construct, and 1.3 and 4.6 for the mCherryFP::BP::mGFP construct) for further studies (data not shown). The expression profile of the two reporter genes was studied in T3 plants by using different tissue samples (seedlings, rosettes, roots and mature seeds). Results confirmed the simultaneous transcription of the pair of genes controlled by the bidirectional promoter in all tested plant tissues, independently of the gene location on the complementary strands. Generally, a higher content of mRNAs derived from the *mGFP* gene than from *mCherryFP* gene was quantified in all tested tissue samples. Root and seed tissues presented the lowest accumulation of transcripts. As expected, plants transformed with the control pmdc107ø vector had basal expression levels of the *mGFP* gene but did not express the *mCherryFP* gene (Supplementary Figure 5).

**FIGURE 4:**
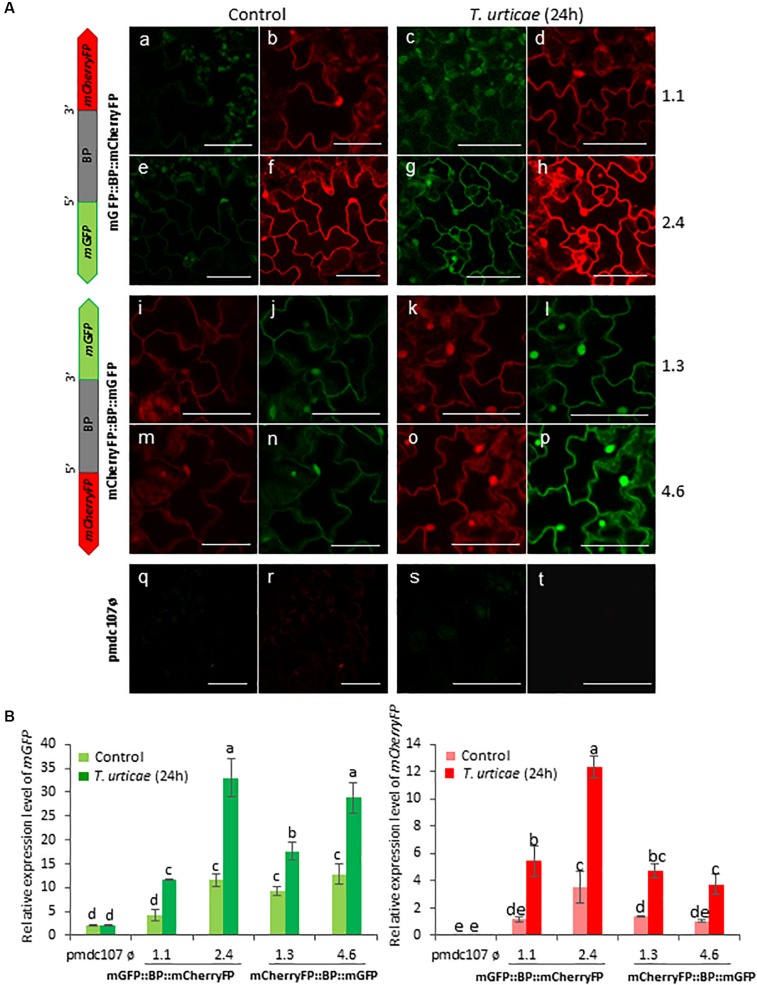
Schematic representation of fusion-constructs and expression assays in stably transformed Arabidopsis plants in response to *T. urticae* infestation. **(A)** Scheme of mCherryFP and mGFP genes under the bidirectional promoter (BD) and confocal stacks spanning Arabidopsis epidermal cells of transformed leaves under control conditions, and after *T. urticae* infestation. Projections are from mCherryFP **(b,d,f,h,i,k,m,o,r,t)** and mGFP **(a,c,e,g,j,l,n,p,q,s)**. Bars are 50 μm. **(B)** Quantitative analyses of *mGFP* and *mCherryFP* gene expression in stably transformed Arabidopsis plants in control, and after 24 h of *T. urticae* infestation. Bars in green and red colors correspond to *mGFP* and *mCherryFP* gene expression, respectively. Data are ±SE of three biological replicates. Different letters indicate statistical differences (*P* < 0.05 Wald χ^2^ followed by LSD test). Arabidopsis transformed lines for mGFP::BP:mCherryFP construct were 1.1 and 2.4, and for mCherryFP:BP::mGFP were 1.3 and 4.6. Primers used for this experiment are listed in Supplementary Table 1.

**FIGURE 5:**
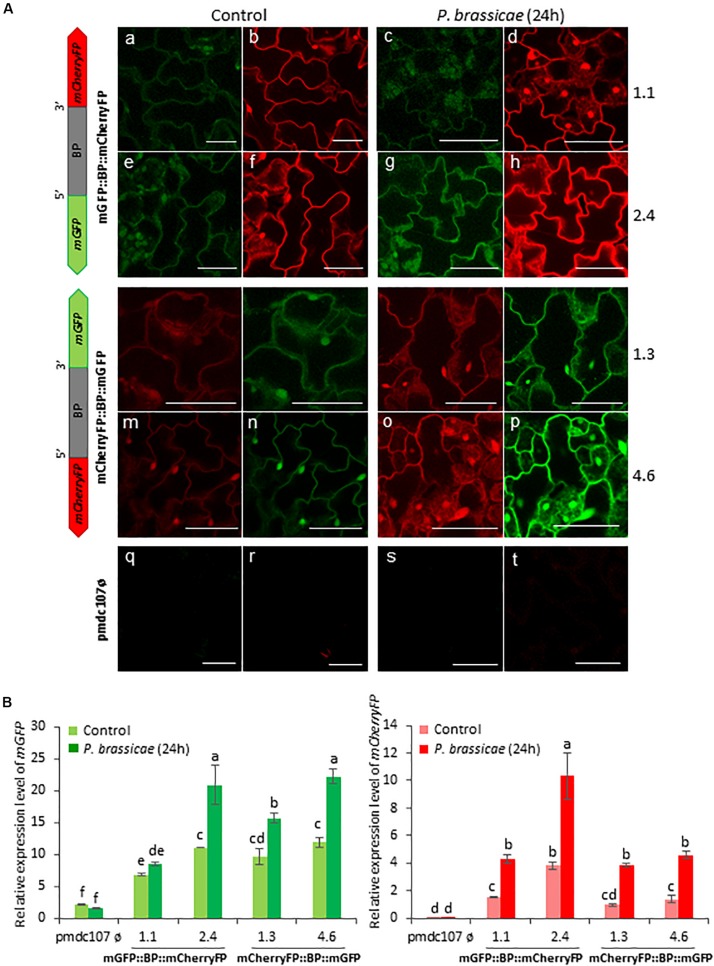
Schematic representation of fusion-constructs and expression assays in stably transformed Arabidopsis plants in response to *P. brassicae* infestation. **(A)** Scheme of mCherryFP and mGFP genes under the bidirectional promoter (BD) and confocal stacks spanning Arabidopsis epidermal cells of transformed leaves under control conditions, and after *P. brassicae* infestation. Projections are from mCherryFP **(b,d,f,h,i,k,m,o,r,t)** and mGFP **(a,c,e,g,j,l,n,p,q,s)**. Bars are 50 μm. **(B)** Quantitative analyses of *mGFP* and *mCherryFP* gene expression in stably transformed Arabidopsis plants in control, and after 24 h of *P. brassicae* infestation. Bars in green and red colors correspond to *mGFP* and *mCherryFP* gene expression, respectively. Data are ±SE of three biological replicates. Different letters indicate statistical differences (*P* < 0.05 Wald χ^2^, followed by LSD test). Arabidopsis transformed lines for mGFP::BP:mCherryFP construct were 1.1, 2.4, and for mCherryFP:BP::mGFP were 1.3 and 4.6. Primers used for this experiment are listed in Supplementary Table 1.

**FIGURE 6:**
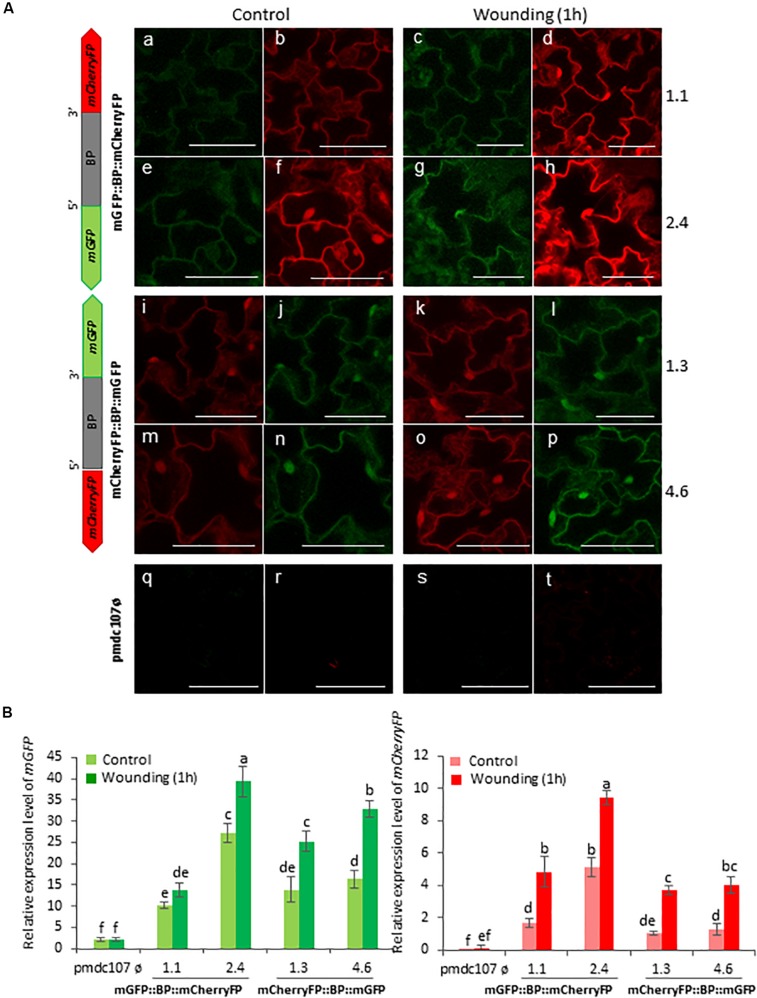
Schematic representation of fusion-constructs and expression assays in stably transformed Arabidopsis plants in response to wounding. **(A)** Scheme of *mCherryFP* and *mGFP* genes driven by the bidirectional promoter (BD) and confocal stacks spanning Arabidopsis epidermal cells of transformed plants after 1 h of wounding. Projections are from mCherryFP **(b,d,f,h,i,k,m,o,r,t)** and mGFP **(a,c,e,g,j,l,n,p,q,s)**. Bars are 50 μm. **(B)** Quantitative analyses of *mGFP* and *mCherryFP* expression by RT-qPCR in Arabidopsis transformed plants after 1 h of wounding. Bars in green and red colors correspond to *mGFP* and *mCherryFP* gene expression, respectively. Data are ±SE of three biological replicates. Differential letters indicate statistical differences (*P* < 0.05 Wald χ^2^ followed by LSD test). Arabidopsis selected lines for mGP::BBP::mCherryFP construct were 1.1. and 2.4, and for mCherryFP::BD::mGFP were 1.3 and 4.6. Primers used for this experiment are listed in Supplementary Table 1.

Arabidopsis transformed plants were used to perform feeding bioassays with *T. urticae* and *P. brassicae* and the expression of the reporter genes was evaluated under the confocal microscope and/or by RT-qPCR analysis after 24^∘^h of infestation. Confocal images showed that the two phytophagous arthropods clearly induced the emission of fluorescence of both reporter genes and in both orientations. No detectable fluorescence was found under the confocal microscope in Arabidopsis transformed with the control vector either in non-infested or in infested plants (Figures 4A, 5A). According to the images, the bidirectional promoter played a coordinated control of the expression of the gene pair, although the intensity of the fluorescence was always stronger when the reporter genes were located at the forward orientation in comparison to the location in the reverse orientation. High levels of fluorescence emitted by Arabidopsis leaves in response to spider mite and caterpillar feeding correlated with increased content of reporter transcripts after infestation (Figures 4B, 5B). Once more, the accumulation of *mGFP* mRNAs was higher than the *mCherryFP* mRNAs, independently on the gene orientation and on the feeding stress. In addition, similar gene expression patterns and comparable confocal images were observed when transformed Arabidopsis plants were mechanically wounded (Figure 6). Likewise, basal expression levels of the *mGFP* gene were observed in the transformed control plants (pmdc107ø), but its expression was not up-regulated by biotic or wounding stress.

**FIGURE 7:**
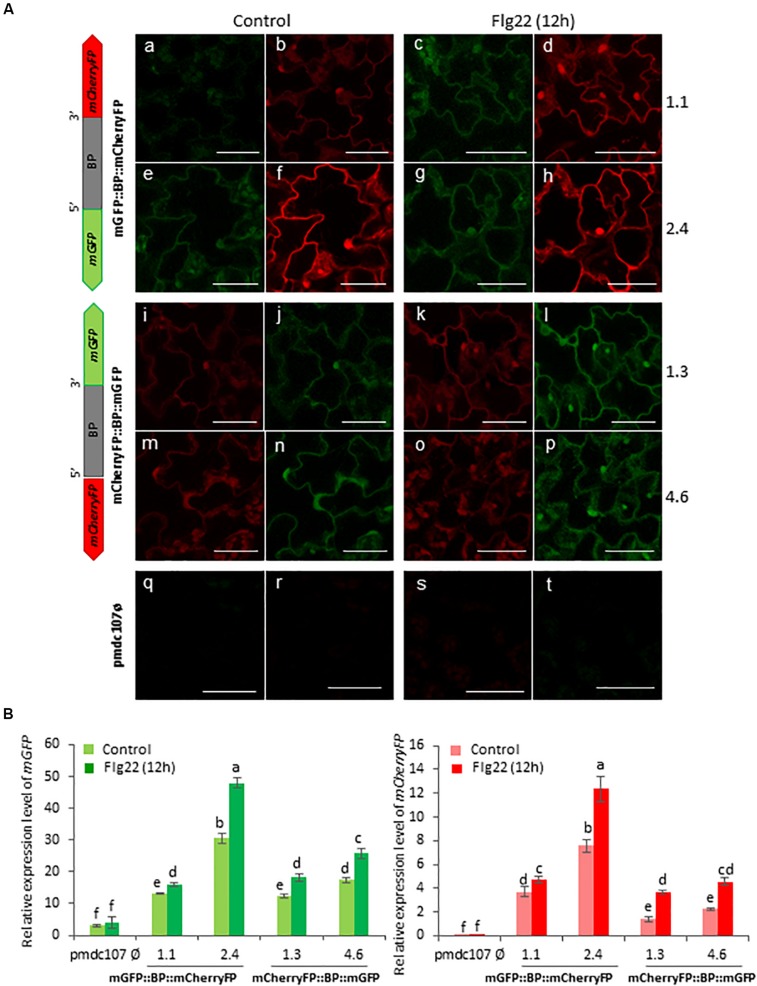
Schematic representation of fusion-constructs and expression assays in stably transformed Arabidopsis plants in response to flagellin treatment. **(A)** Scheme of *mCherryFP* and *mGFP* genes driven by the bidirectional promoter (BD) and confocal stacks spanning Arabidopsis epidermal cells of transformed plants after 1^∘^h offlagelin22 (Flg22) treatment. Projections are from mCherryFP **(b,d,f,h,i,k,m,o,r,t)** and mGFP **(a,c,e,g,j,l,n,p,q,s)**. Bars are 50 μm. **(B)** Quantitative analyses of *mGFP* and *mCherryFP* expression by RT-qPCR in Arabidopsis transformed plants after 1 h of Flg22 treatment. Bars in green and red colors correspond to *mGFP* and *mCherryFP* gene expression, respectively. Data are ±SE of three biological replicates. Differential letters indicate statistical differences (*P* < 0.05 Wald χ^2^ followed by LSD test). Arabidopsis selected lines for mGP::BBP::mCherryFP construct were 1.1. and 2.4, and for mCherryFP::BD::mGFP were 1.3 and 4.6. Primers used for this experiment are listed in Supplementary Table 1.

**FIGURE 8:**
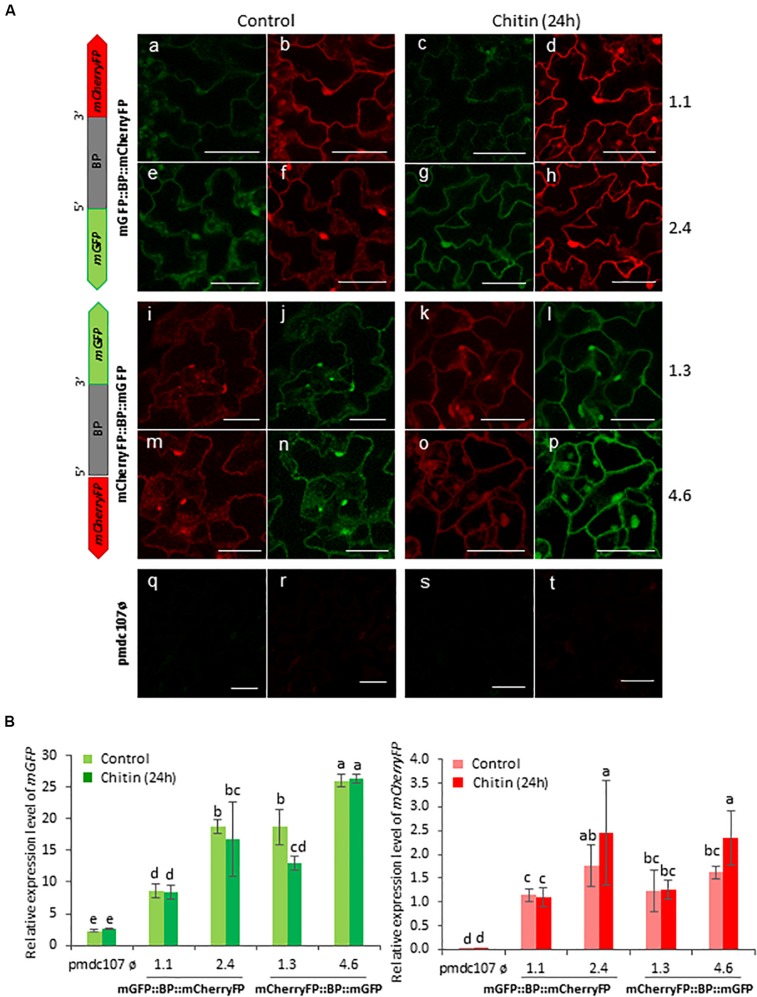
Schematic representation of fusion-constructs and expression assays in stably transformed Arabidopsis plants in response to Chitin treatment. **(A)** Scheme of *mCherryFP* and *mGFP* genes driven by the bidirectional promoter (BD) and confocal stacks spanning Arabidopsis epidermal cells of transformed plants after 1 h of Chitin treatment. Projections are from mCherryFP **(b,d,f,h,i,k,m,o,r,t)** and mGFP **(a,c,e,g,j,l,n,p,q,s)**. Bars are 50 μm. **(B)** Quantitative analyses of *mGFP* and *mCherryFP* expression by RT-qPCR in Arabidopsis transformed plants after 1 h of chitin treatment. Bars in green and red colors correspond to *mGFP* and *mCherryFP* gene expression, respectively. Data are ±SE of three biological replicates. Differential letters indicate statistical differences (*P* < 0.05 Wald χ^2^ followed by LSD test). Arabidopsis selected lines for mGP::BBP::mCherryFP construct were 1.1. and 2.4, and for mCherryFP::BD::mGFP were 1.3 and 4.6. Primers used for this experiment are listed in Supplementary Table 1.

Arabidopsis plants were also treated with two elicitors, flagellin and chitin, and showed a differential response to each one. The Flg22 elicited the expression of the two reporter genes as well as the emission of the fluorescence under confocal microscope as the biotic and wounding treatments did (Figure 7). In contrast, no significant differences were detected after the chitin treatment between treated and non-treated plants (Figure 8). RT-qPCR assays of the two divergent genes, *At5g10290* and *At5g10300*, were also carried out in Arabidopsis rosettes after *T. urticae* and *P. brassicae* feeding, as well as under wounding, Flg22 and chitin treatments. Results corroborated that both genes were simultaneously transcribed and induced by all treatments except by chitin (Supplementary Figure 6).

Since both, the reporter genes under the control of the bidirectional promoter and the divergent *At5g10290* and *At5g10300* genes, were up-regulated in response to pest feeding, wounding and Flg22 treatments but not with chitin, defense signaling pathways in transgenic plants and in T-DNA insertion lines were tested. The expression levels of three genes associated with hormonal pathways such as the plant defensin *PDF1.2*, the vegetative storage *VSP2* and the pathogenesis related *PR1*, involved in ethylene/jasmonic acid (JA) and salicylic acid (SA) responses, were quantified. Interestingly, the *PDF1.2* gene was highly over-expressed in response to spider mite in the Arabidopsis lines transformed with the bidirectional promoter in contrast to the no significant induction quantified in the plants transformed with the control pmdc107ø vector. Besides, mite infestation induced the expression of *VSP2* and *PR1* genes in all tested plants. In contrast, the induction of *VSP2* and *PDF1.2* genes mediated by *P. brassicae* feeding was observed in all Arabidopsis genotypes while the *PR1* gene was always down-regulated after this biotic stress (Supplementary Figure 7). Regarding the wounding and elicitor treatments, the *PDF1.2* gene was higher induced by Flg22 in plants with the fusion-constructs than in control plants, while was similarly induced by wounding and chitin in all tested plants, independently of the bidirectional promoter presence (Supplementary Figure 8). In addition, the expression profiles of *VSP2* and *PR1* genes showed a up-regulation mediated by the three treatments, with few differences among all Arabidopsis genotypes (Supplementary Figure 8). Remarkably, the basal transcript content of *PDF1.2*, *VSP2*, and *PR1* genes in the two Arabidopsis T-DNA insertion lines did not show significant changes in comparison to the expression levels quantified in Col-0 plants (Supplementary Figure 9).

## Discussion

Gene pairs oriented in divergent transcriptional configuration and controlled by bidirectional promoters are abundant in plant genomes. In Arabidopsis, more than 13% of its genes are driven by bidirectional promoters (Wang et al., 2009), but the biological relevance of these intergenic regions has been only studied in some cases (Mitra et al., 2009; Kourmpetli et al., 2013; Pratibha et al., 2013; Mishra and Grover, 2014; Liu et al., 2015; Sharma et al., 2017).

Making a search of up-regulated genes in response to spider mite feeding, we identified two over-expressed divergent genes, *At5g10290* and *At5g10300*, separated by an intergenic region. Here, it is proven that the 645 bp intergenic sequence between the transcription start sites of both genes corresponds to a bidirectional promoter. The *At5g10290* gene encoded a LRR receptor kinase that could be involved in the perception of molecules derived from phytophagous and/or pathogens initiating the plant transduction defense pathway. The *At5g10300* gene encoded a hydroxynitrile lyase that catalyze an enzymatic reaction that release hydrogen cyanide, a toxic compound against herbivores and pathogens (Zhu et al., 2015). The gene pair potentially involved in plant defense, was simultaneously transcribed in the same tissues but the promoter strength was asymmetric as was shown by the differential levels of gene expression quantified by RT-qPCR assays. The asymmetry might be caused by the presence of conserved *cis*-acting elements in the opposite strands with potential antagonistic or synergistic roles to regulate the transcription (Liu et al., 2015). Co-expression does not necessarily mean identical spatial and temporal expression or equal responses to stresses. A coordinated expression of genes to overcome abiotic and biotic cues is regulated by the *cis*-motifs contained in the promoter. Thus, T-DNA insertions disturbing the bidirectional promoter architecture presented a negative effect on the gene responses, and subsequently on the damage produced by *T. urticae* feeding. Since both insertion lines caused the silencing of the opposite genes, the transcriptional regulation most likely involve the whole promoter region. Sequences upstream T-DNA insertion points control both, the nearest and the farthest genes, which reinforces the idea of a strongly coordinated response against a specific stress.

In this context and as many authors previously did, two reporter genes were fused to the promoter in both orientations, for a deep analysis of its function *in planta*. In previous studies, *GUS* and *GFP* genes were the reporters most commonly used but the *GUS* gene requires an enzymatic reaction to generate the reporter product (Mitra et al., 2009; Wang et al., 2016; Oropeza-Aburto et al., 2017; Yang et al., 2018). This step implies not only a cost and time-consuming but also a time lag between the two reporter quantification assays. To avoid it, enhanced variants of *GFP* and *CherryFP* genes encoding fluorescent proteins with different excitation/emission wavelengths were selected here for a rapid gene co-expression analysis *in vivo*. Transient and stable transformation assays revealed that the two genes coordinately regulated their transcripts and the fluorescent emissions, in terms of constitutive as well as induced expression profiles in response to biotic treatments. The accumulation of *mGFP* mRNAs was always higher than the *mCherryFP* mRNA content, independent on the gene orientation, tissue sample and stress treatment, probably due to a major stability of *mGFP* transcripts. However, gene expression data correlated to the fluorescence observed under the microscope for both reporters.

Interestingly, confocal images and mRNA expression patterns clearly indicated that the bidirectional promoter was inducible by *T. urticae* and *P. brassicae* feeding as well as by wounding. Wounding, although is not considered a biotic stress, is an additional effect produced as consequence of the feeding process. These results were in agreement with the up-regulated expression of the pair of genes located on the opposite promoter strands in the resistant Bla-2 Arabidopsis accession, after mite infestation (Zhurov et al., 2014). In addition, the fluorescence emission of both genes in infested Arabidopsis plants corroborated the asymmetrical character of the promoter, with stronger activity in the forward than in the reverse orientation.

There are previous examples of bidirectional promoters whose activity was induced by biotic stresses. Shin et al. (2003) reported the up-regulation of the *GUS* gene controlled by a bidirectional promoter from hot pepper in response to Tobacco Mosaic Virus infection, in transgenic tobacco plants. Following the same experimental strategy, Banerjee et al. (2013) showed the induction of the GUS staining and the enzymatic GUS activity driven by an Arabidopsis bidirectional promoter when transformed tobacco plants were inoculated with spores from *Peronospora tabacina.* The main difference between these results and the data presented here is that in the mentioned examples only one GUS-construct with the reporter gene in one orientation was checked. Our findings demonstrated the simultaneous induction of the two reporter genes located in both orientations of the bidirectional promoter mediated by arthropod phytophagous. In addition to the promoter inducibility produced by pests, the pathogen elicitor flagellin also provoked the up-regulation of the reporter genes.

The coordinated function of a bidirectional promoter has to be achieved by the control of the regulatory motifs located in its opposite strands. Computational analysis evidenced the existence of *cis*-elements potentially recognized by TFs within the intergenic region, mainly from the DOF family but also from the AP2/ERF, Golden 2-like and bHLH families. Most of these motifs are commonly observed in promoters of genes encoding defense responses to pathogen infection and insect attack (Barah et al., 2013; Amorim et al., 2017). Interestingly, putative DOF-binding sequences were particularly abundant in this bidirectional promoter, with six elements close located among them. Although this TF family is not described as the most relevant in defense to biotic stresses, the functional validation of the role played by DOFs in response to pest attack has been shown in the case of the AtDof1.1. This TF, also known as OBP2, was up-regulated by *Spodoptera littoralis* and methyl jasmonate. It took part of the network involved in the biosynthesis of important defense compounds such as glucosinolates, characteristics of the Brassicaceae family (Skirycz et al., 2006). Likewise, Skirycz et al. (2007) reported that the AtDOF4.2 altered the flavonoid and the hydrocinnamic acid synthesis, which potentially modified the suberin production, an important component of the physical plant barriers with protective roles. In contrast, the importance of the bHLH and AP2/ERF TFs in the regulation of plant immunity to pathogens and insect herbivory is widely reported (Tsuda and Somssich, 2015). Notably, whereas ethylene response factors (ERF) are integrators of hormonal pathways and participate in the transcriptional regulation of JA/ethylene-responsive defense genes (Huang et al., 2016), the basic helix-loop-helix (bHLH) factors are mainly related to the control of different subsets of the JA-dependent transcriptional defense responses (Fernandez-Calvo et al., 2011; Schweizer et al., 2013). Finally, Golden 2-like TFs have been related to the defense against pathogens affecting in some way SA and JA signaling regulatory network (Chen et al., 2016). In consequence, a complex setup of defenses is achieved through the binding to specific responsive elements contained in the promoter sequence, controlled by a hormonal crosstalk with a pivotal role in the orchestration of defenses.

The complexity in the regulation of this network is further supported by the differential over-expression of defense products derived from JA, Et and SA biosynthesis pathways. Whereas *VSP2* and *PR1* genes were similarly induced by most of the stresses in plants transformed with the fusion-construct or with the control vector, the expression of *PDF1.2* after spider mite and Flg22 treatments was significantly higher when the construct harboring the promoter and the reporter genes was present. These alterations in *PDF1.2* gene expression were not due to a positional effect of the transgenes since expression patterns were similar between independent transgenic lines. Most likely, the presence of an additional copy of the bidirectional promoter may be in some way modifying the final response associated to the hormonal signaling pathways. This effect cannot be directly attributed to an altered regulation of the endogenous bidirectional promoter caused by the presence of the additional copy. Since the expression of the flanking *At5g10300* and *At5g10290* genes was similar to that observed in the control line in response to any biotic treatment, hormonal signaling alterations may be probably associated to indirect effects such as the scavenging of transcriptional factors important for a full hormonal response. Interestingly, the basal expression levels of the three hormone-related genes were similar in the two T-DNA insertion lines than in the Col-0 plants, which indicates that the relevance of the bidirectional promoter in the regulation of hormonal signaling pathways relies in the presence of the biotic stress. In the case of the plant response to spider mites, alterations in the expression of *PDF1.2* suggest that the ethylene/JA branch-signaling pathway can be involved in the regulation of the Arabidopsis gene pair controlled by the bidirectional promoter to generate defenses. Accordingly, it has been already reported that JA is an essential hormone, but not the unique, for establishing the mite-induced defense responses (Zhurov et al., 2014; Santamaria et al., 2017, 2019).

The relevant finding emerging from this study is that two genes involved in defense against biotic stresses, particularly in the response to spider mite feeding, are packed together, induced by different biotic stresses and simultaneously controlled by a common regulatory mechanism. The bidirectional promoter is the key element in such an efficient defense mechanism and it could be the base to design regulatory modules with the potential to express multigene traits in a coordinately regulated manner.

## Data Availability

All datasets for this study are included in the manuscript and the Supplementary Files.

## Author Contributions

ID conceived the research. AA and MES performed most of the experimental research. MM performed the bioinformatics analyses. PG-M helped with the microscopic data. All authors participated in the design, acquisition, analysis, and interpretation of the data for the work, and contributed to the final version of the manuscript.

## Conflict of Interest Statement

The authors declare that the research was conducted in the absence of any commercial or financial relationships that could be construed as a potential conflict of interest.

## Footnotes

1 http://arabidopsis.info/BasicForm

2 http://bioinfo.ut.ee/primer3-0.4.0/
