# Supplementary material for: Plant Defenses Against Pests Driven by a Bidirectional Promoter
Source: Front Plant Sci. 2019 Jul 17;10:930. doi: 10.3389/fpls.2019.00930 (PMC6652247; doi:10.3389/fpls.2019.00930)
Supplement: Supplementary file 1 [file Data_Sheet_1.pdf]

Supplementary Material

Plant defenses against pests driven by a bidirectional promoter

Arnaiz A, Martinez M, Gonzalez-Melendi P, Grbic V, Diaz I, Santamaria ME*
*Correspondence: M. Estrella Santamaria. E-mail: me.santamaria@upm.es

1.Supplementary Figures and Tables

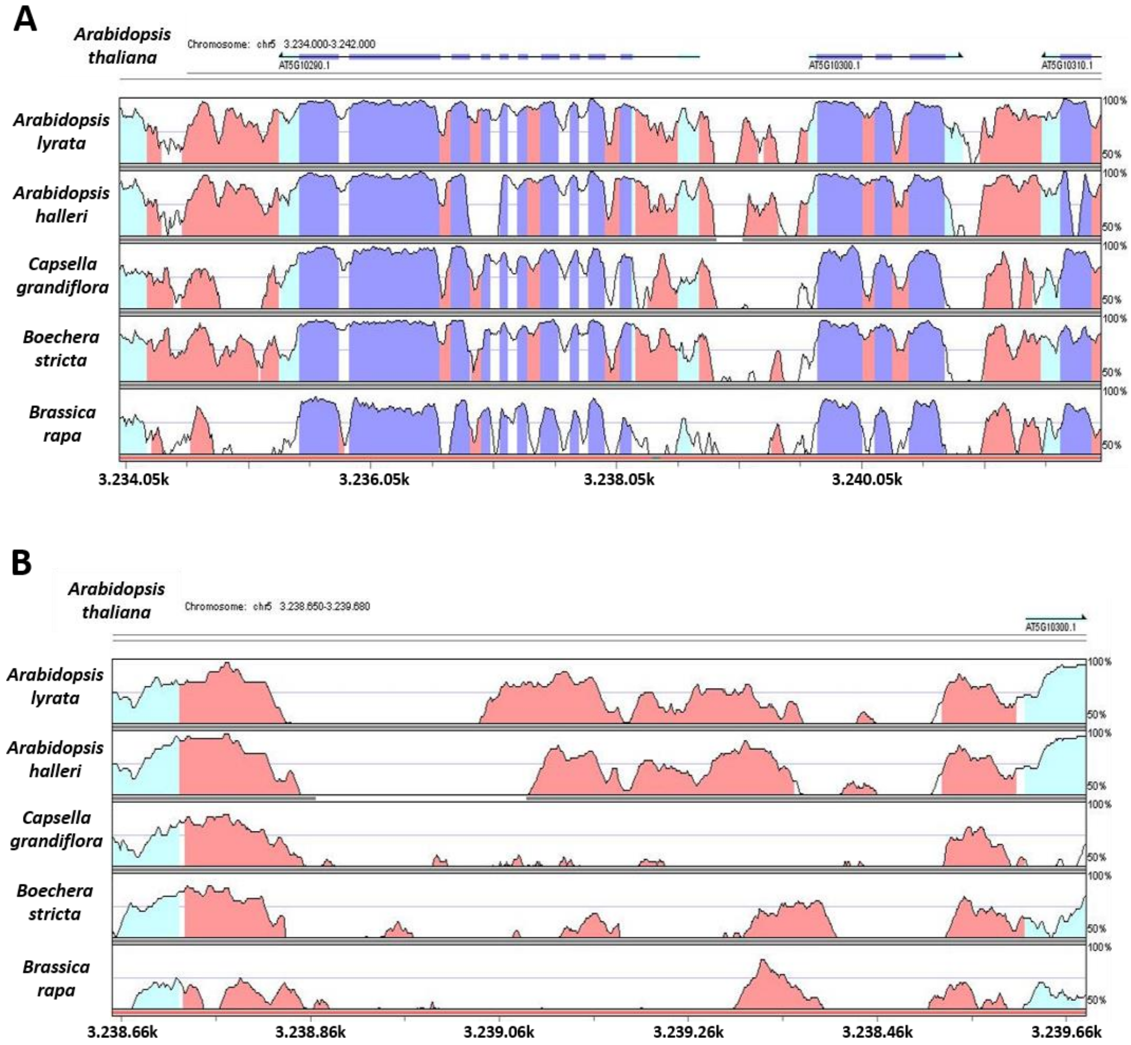

Supplementary Figure 1. The VISTA plots of pair-wise comparisons of different Brassicaceae species to *A. thaliana* as generated by the mVISTA program. **(A)** Genomic regions spanning *At5g10290* and *At5g10300* genes. **(B)** Genomic region spanning putative bifunctional promoter between *At5g10290* and *At5g10300* genes. Intron and exon annotation of *A. thaliana* is shown on top (exons are represented by thick lines, introns by thin lines) and filled portions of the graphs indicate conservation of more than 70% with a width of at least 100 bp (red for intron and intergenic sequences, light blue for 5'UTR and 3'UTR sequences, dark blue for exon sequences). Numbers at the bottom of the plots indicate positions relative to the *A. thaliana* chromosome 5.

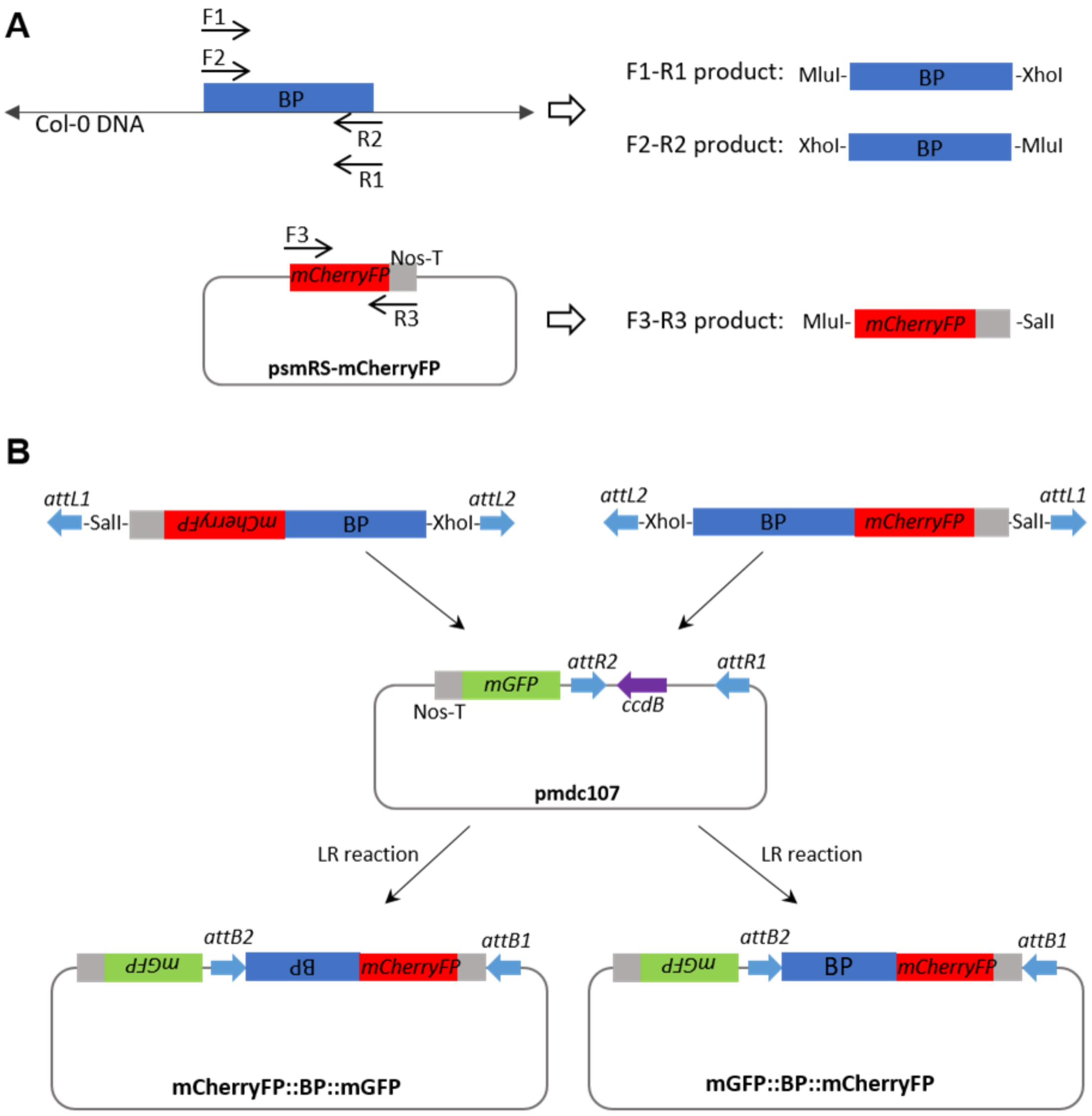

Supplementary Figure 2. Scheme of the design to prepare constructions containing the bidirectional promoter fused to two fluorescence reporter genes. **(A)** Intergenic region (bidirectional promoter: BP) was amplified using two pairs of primers (F1 and R1, and F2 and R2). The *mCherryFP* gene with the Nos terminator was amplified from the psmRS-mCherryFP vector using the pair of primers (F3 and R3). **(B)** All DNA fragments were purified, digested and ligated into the pENTRY3C vector. LR reactions were conducted with pmdc107 vector by homologous recombination to get the constructs mCherryFP::BP::mGFP and mGFP::BP::mCherryFP. Primers are indicated in Supplementary Table 1.

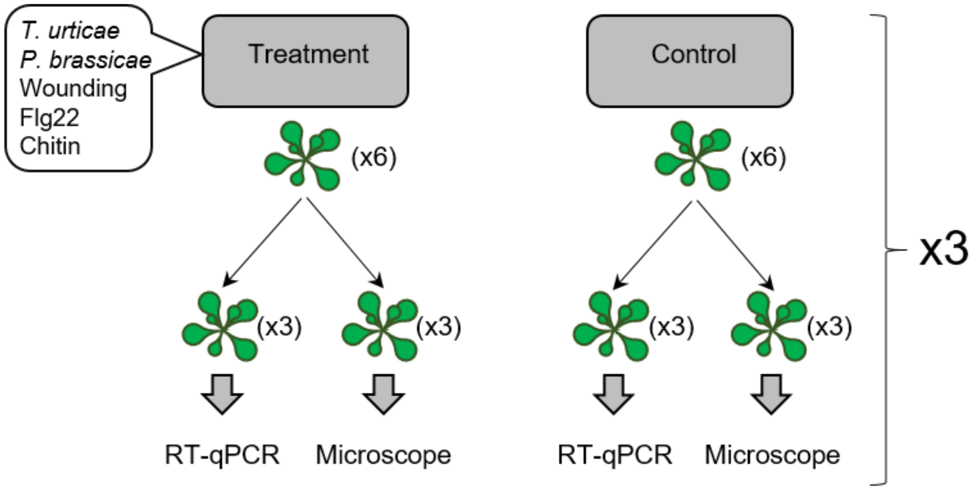

Supplementary Figure 3. Scheme of experimental design of feeding assays and treatments. Twelve plants (three week-old) were treated (six plants) or non-treated (six plants) for each transgenic line (pmdc107ø, mCherryFP::BP::mGFP, mGFP::BP::mCherryFP). After treatments, three plants for each genotype were collected and frozen in liquid N_2 for RT-qPCR or were observed under the microscope. All experiments were performed in triplicate.

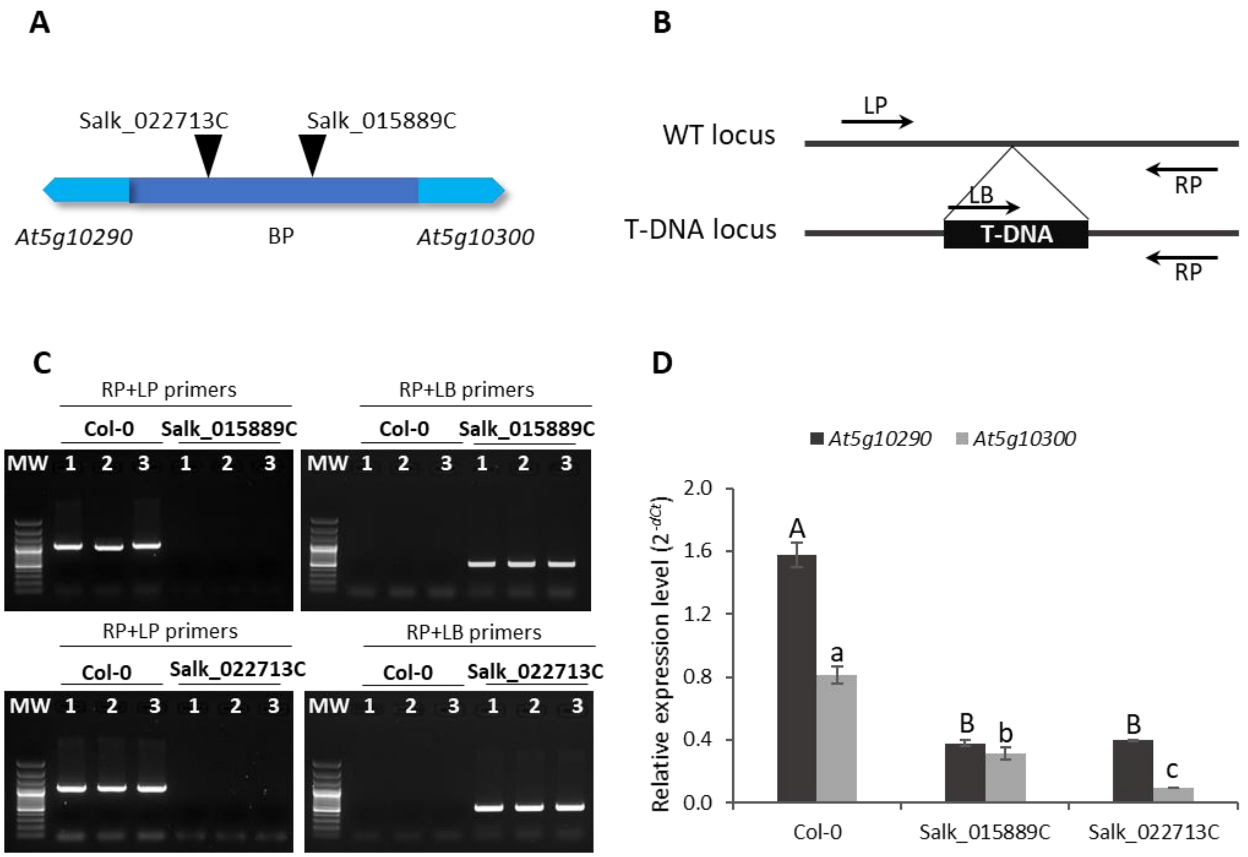

Supplementary Figure 4. Molecular characterization of Arabidopsis mutant lines. **(A)** Scheme of the position of the T-DNA insertions (arrowhead) in the promoter T-DNA insertion lines (Salk_022713C and Salk_015889C). **(B)** Location of the primers used for validation of Salk lines. **(C)** PCR assays of Salk lines to show homozygous status. **(D)** Expression levels of *At5g10290* and *At5g10300* genes in Col-0 and Salk lines. Gene expression, referred as relative expression level to ubiquitin (2^{-dCt}). Data are means ±SE of three replicates. Different letters indicate significant differences (($P<0.05$ Wald χ^2 followed by LSD test). Primers used for these assays are listed in Supplementary Table 1.

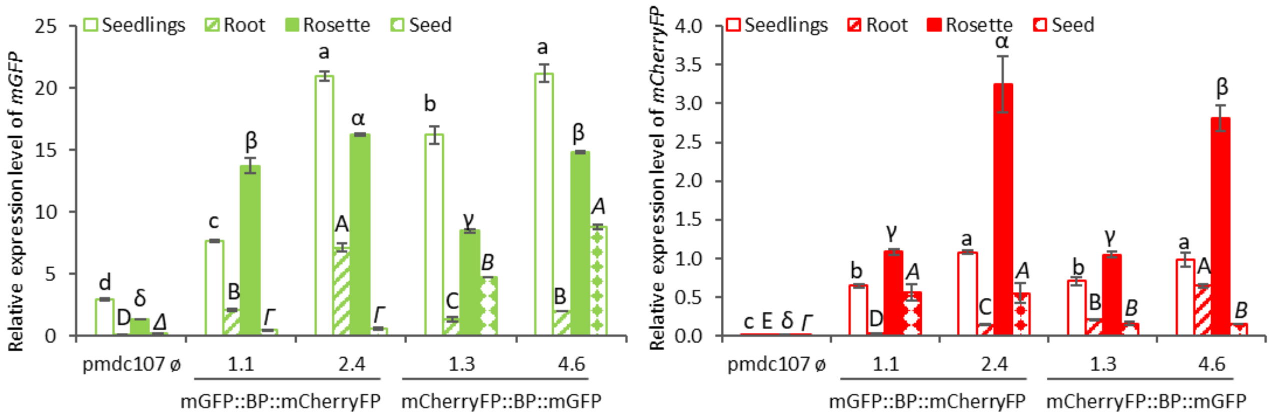

Supplementary Figure 5. Expression profiles of *mGFP* and *mCherryFP* reporter genes in Arabidopsis stably transformed plants with the fusion-constructs and the control vector, by RT-qPCR. Relative expression levels of reporter genes in different tissues (seedlings, rosettes, roots and seeds). Green and red colours correspond to *mGFP* and *mCherryFP* gene expression, respectively. Control vector: pmdc107ø. Fusion-constructs: mGFP::BP::mCherryFP (lines 1.1 and 2.4) and mCherryFP::BP::mGFP (lines 1.3 and 4.6). Different letters indicate statistical differences (P<0.05 Wald χ^2 followed by LSD test).

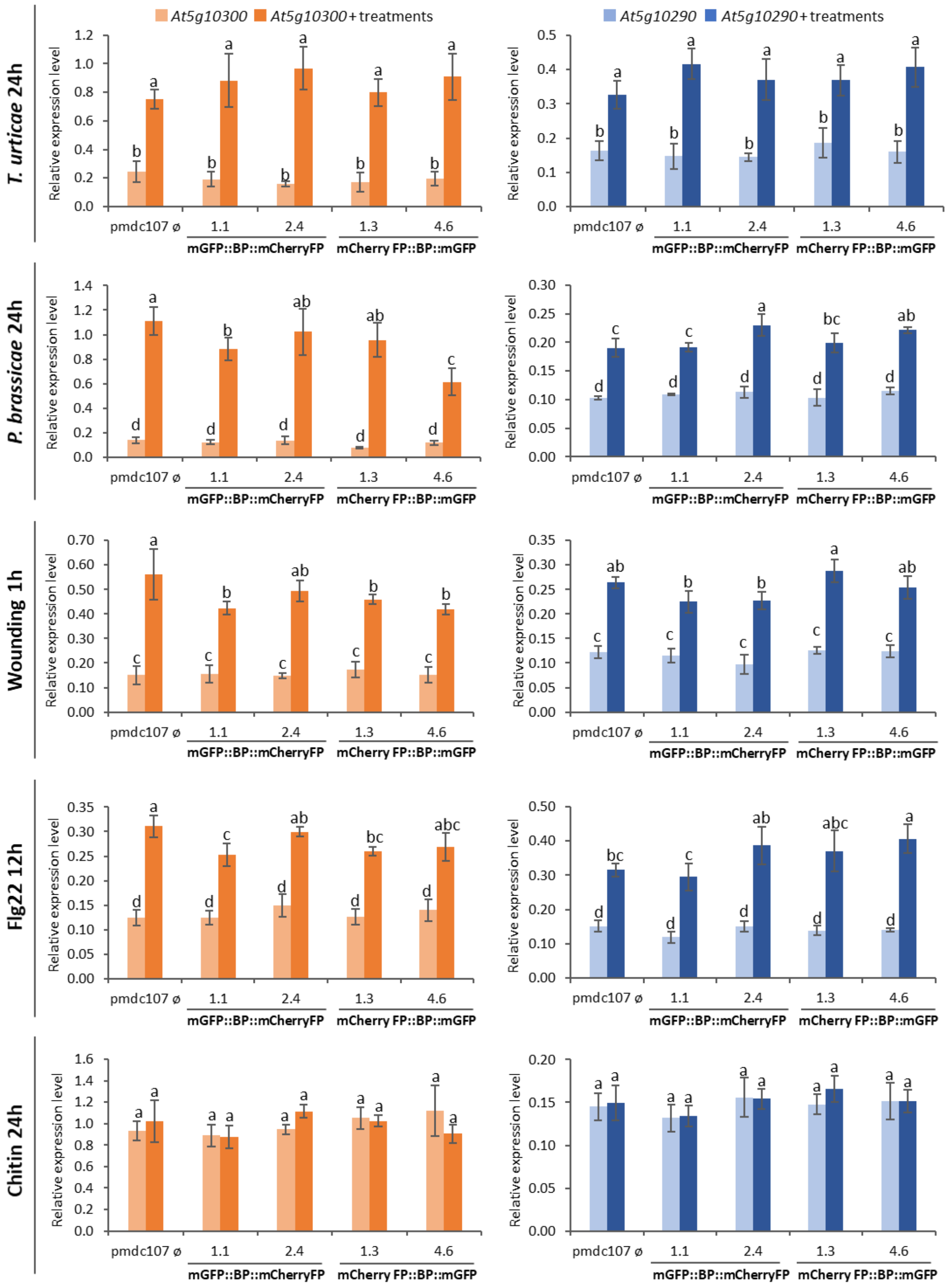

Supplementary Figure 6. Expression levels of *At5g10290* and *At5g10300* divergent genes in stably transformed plants after *T. urticae* and *P. brassicae* feeding and after wounding, Flg22 and Chitin treatments. Gene expression is referred as relative expression level to ubiquitin (2^{-dCt}). Data are means ±SE of three biological replicates. Different letters indicate significant differences (P<0.05 Wald χ^2 followed by LSD test). Arabidopsis selected lines for mGFP::BP::mCherryFP construct were 1.1. and 2.4, and for mCherryFP::BP::mGFP were 1.3 and 4.6.Primers used are listed in Supplementary Table 1.

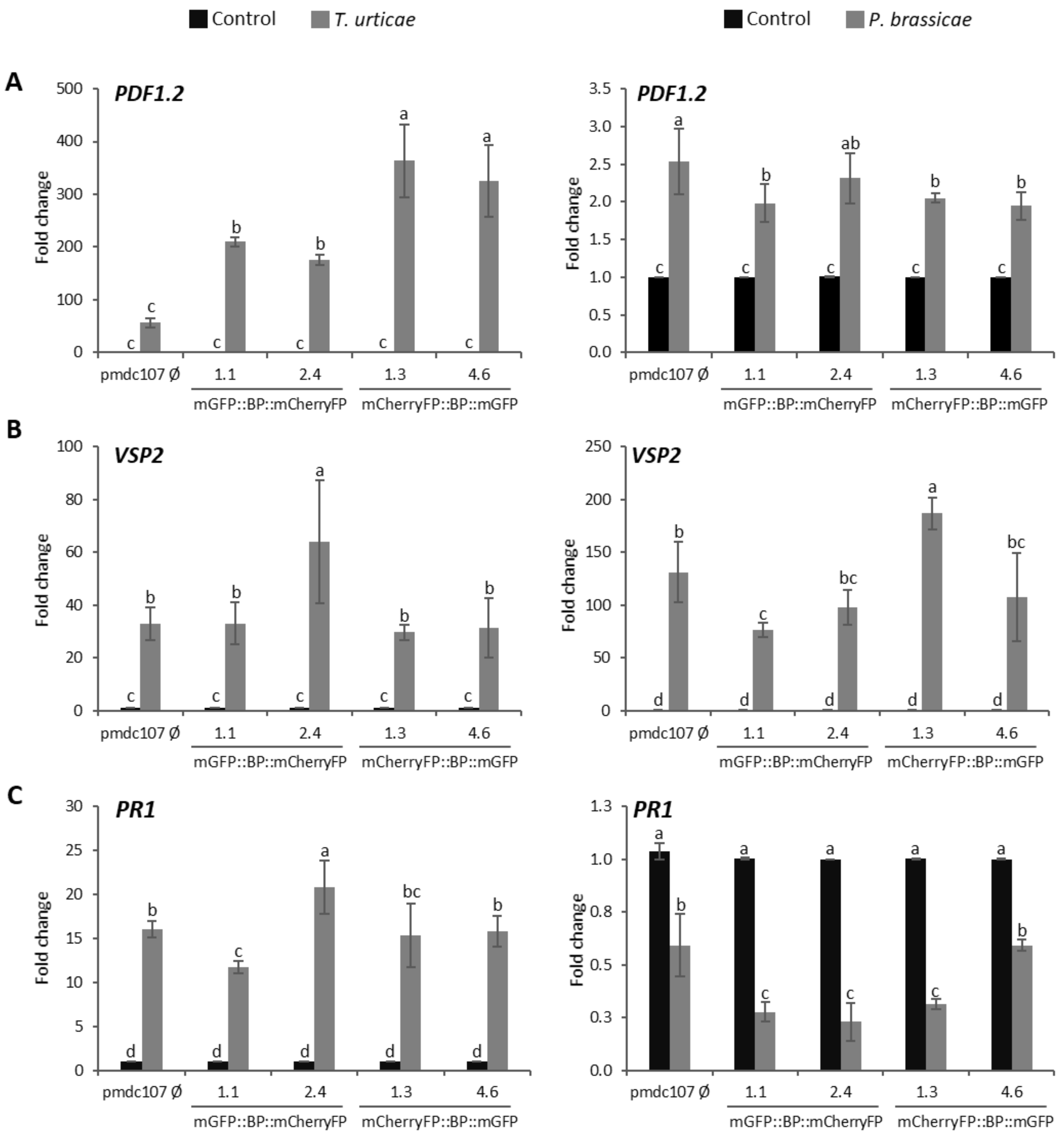

Supplementary Figure 7. Expression levels of hormone-related genes after 24h of arthropod infestation. **(A)** Plant defensin 1.2 gene (*PDF1.2*). **(B)** Vegetative storage protein 2 gene (*VSP2*). **(C)** Pathogenesis related-gene 1 (*PR1*). Left and right panels correspond to *T. urticae* and *P. brassicae* infestation, respectively. Gene expression, referred as fold change (2^{-ddCt}) was calculated using the control (no infestation) for each transformed line as an internal calibrator. Data are means ±SE of three replicates. Different letters indicate significant differences (P<0.05 Wald χ^2, followed by LSD test). Primers are indicated in Supplementary Table 1.

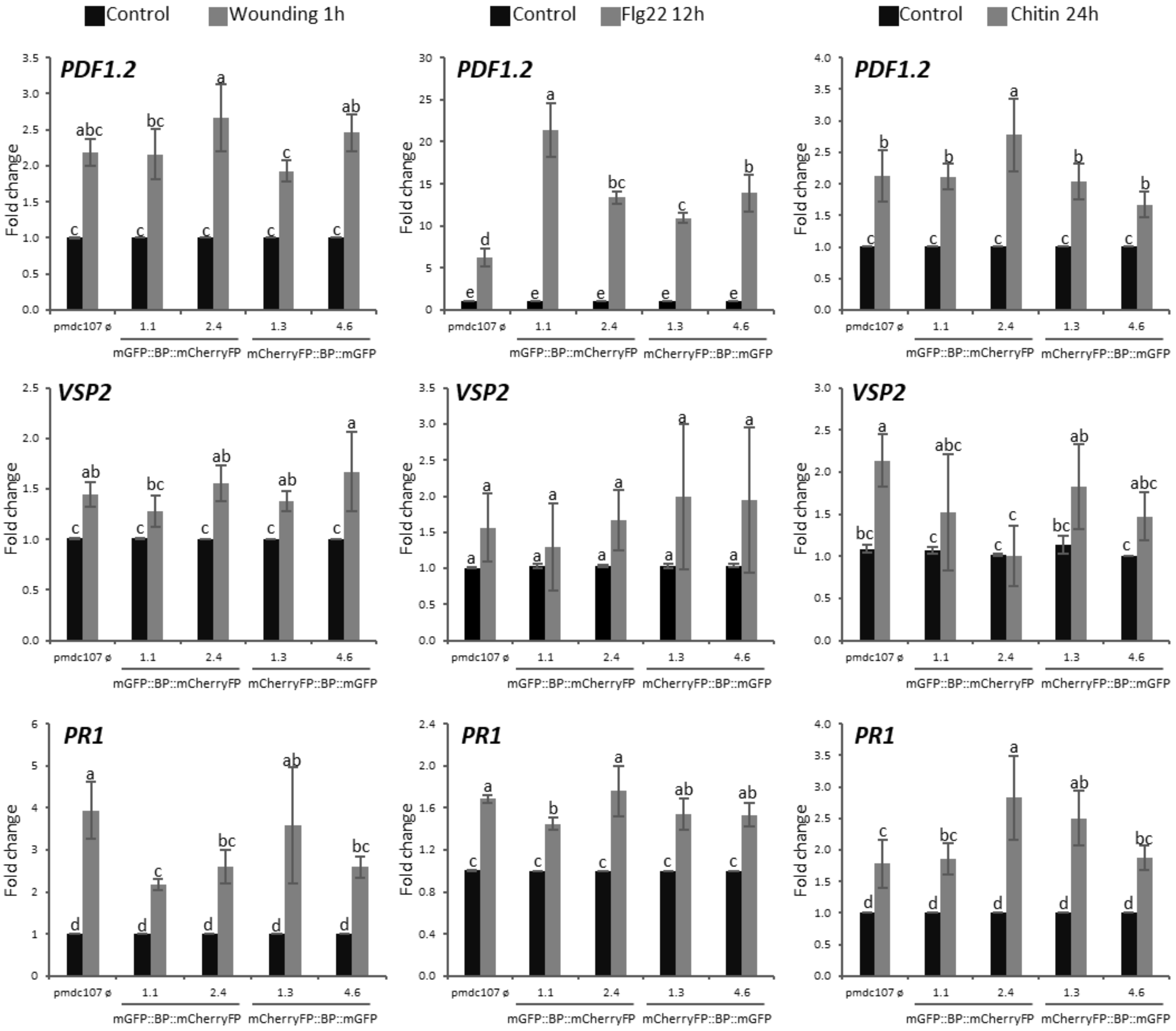

Supplementary Figure 8. Expression levels of hormone-related genes after 1h of mechanical wounding, 12h of Flg22 treatment and 24h of chitin treatment. **(A)** Plant defensin 1.2 (*PDF1.2*). **(B)** Vegetative storage protein 2 (*VSP2*). **(C)** Pathogenesis related-gene 1 (*PR1*). Left panels correspond to mechanical wounding treatment, and central and right panels correspond to Flg22 and chitin treatments, respectively. Gene expression, referred as fold change (2^{-ddCt}) was calculated using the control (no treatment) for each transformed line as an internal calibrator. Data are means ±SE of three replicates. Different letters indicate significant differences (P<0.05 Wald χ^2, followed by LSD test). Primers are indicated in Supplementary Table 1.

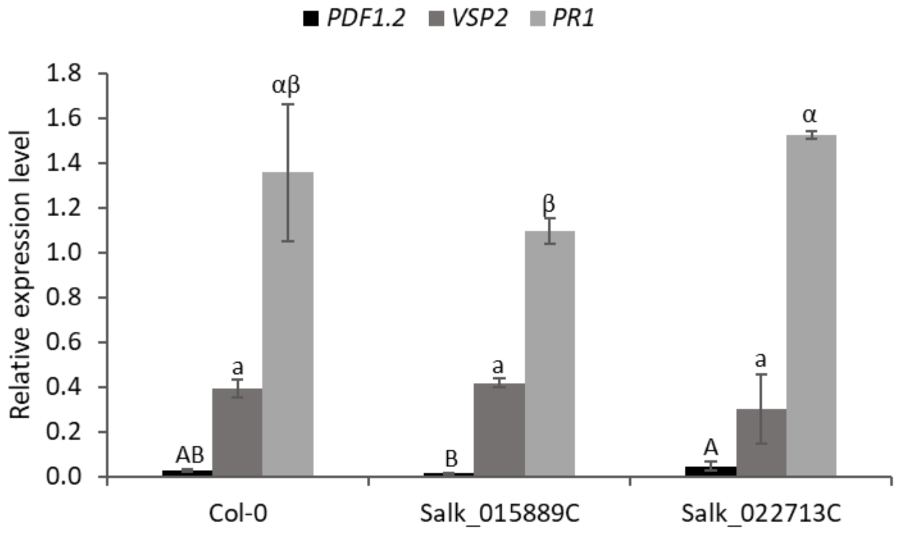

Supplementary Figure 9. Expression levels of hormone-related genes (Plant defensin 1.2 (*PDF1.2),* Vegetative storage protein 2 (*VSP2)* and Pathogenesis related-gene 1 (*PR1*)) in Col-0 and two T-DNA insertion lines (Salk_ 015889C and Salk_022713C). Gene expression, referred as relative expression level to ubiquitin (2^{-dCt}). Data are means ±SE of three biological replicates. Different letters indicate significant differences (P<0.05 Wald χ^2 followed by LSD test). Primers used are listed in Supplementary Table 1.

Supplementary Table 1. Sequences of oligonucleotide used for qRT-PCR, PCR-cloning and validation of T-DNA insertion in the Salk lines.

Gene	Primer name	Sequence (5' → 3')	Purpose
Ubiquitin	UBQ-F	GCTCTTATCAAAGGACCTTCGG	RT-qPCR - Gene expression assays
	UBQ-R	CGAACTTGAGGAGGTTGCAAAG	
AT5G10290	At5g10290-F	ACGTGAAGAAACTGGAAAGA	
	At5g10290-R	AGCAAAGCCACTTGTATCAT	
AT5G10300	At5g10300-F	GGAGGTTTGGGAGATTGTGA	
	At5g10300-R	GACCCTTGCCTATGCAACAT	
AT5G44420	PDF1.2-F	GTTCTCTTTGCTGCTTTCGAC	
	PDF1.2-R	GCAAACCCCTGACCATGT	
AT5G24770	VSP2-F	ATGCCAAAGGACTTGCCCTA	
	VSP2-R	CGGGTCGGTCTTCTCTGTTC	
AT2G14610	PR1-F	TCAGTGAGACTCGGATGTGC	
	PR1-R	CGTTCACATAATTCCCACGA	
AY179605 (Actin)	Actin-F	GATGGACAAGTCATCACCATTG	
	Actin-R	CTGAGGACAATGTTTCCGTACA	
mGFP	mGFP-F	CCTGTCCTTTTACCAGACAACC	
	mGFP-R	ATCCCAGCAGCTGTTACAAACT	
mCherryFP	mCherryFP-F	CAACATCAAGTTGGACATCACC	
	mCherryFP-R	TACTTGTACAGCTCCTCCATGC	
T-DNA	LB	GCAATCAGCTGTTGCCCGTCTCACTGGTG	PCR - T-DNA insertion validation
	Salk_015889C LP	ACACGAACATGCTCAATTTCC	
	Salk_015889C RP	TTACTAGTCTCCTCGGTCGGC	
	Salk_022713C LP	GGAAGAGATTTGAGGGTTTCG	
	Salk_022713C RP	TCCGTGCAACTTGTAATTTCC	
Intergenic region. Bidirectional promoter	(MluI)BP- F (F1)	ACGCGTCTCATCTCATTAATTG	PCR-Cloning
	BP(XhoI)-R (R1)	CTCGAGATGTCTCCGTAGAC	
	(XhoI)BP-F (F2)	CTCGAGCTCATCTCATTAATTG	
	BP(MluI)- R (R2)	ACGCGTATGTCTCCGTAGACG	
mCherryFP	(MluI)mCherryFP-F (F3)	ACGCGTATGGTGAGCAAGGG	
	mCherryFP-NosT(SalI)-R (R3)	GTCGACCCGATCTAGTAAC	
